# Trans. Pennsylvania Med. Society

**Published:** 1871-12

**Authors:** 


Transactions of the Medical Society of the State of Pennsylvania, at its Twenty-second Annual Session, held at Williamsport, June, 1871. Collins, printer, 705 Jayne street, Philadelphia.

This is a volume of 467 pages, the greater part of the contents being made up of the reports of the various county medical societies made to the State Society. These reports embody a large amount of valuable and interesting information in regard to their respective localities. A certain form is given for the making out of these reports, which has been followed in the main, producing a uniformity very desirable for purposes of comparison. The following are the leading topics considered in each: 1st. Causes which modify the Health of the County, such as Locality, Hydrography or Drainage' Topography, Meteorology. 2nd. Mortality Tables. 3rd. Prevalent Diseases, Epidemics and Endemics of the Year, Fevers, etc.

In addition to these reports the following papers are published: "A Case of Thyroid Dislocation of the Hip-joint in the second stage of Coxalgia; Reduction by Manipulation, by Benjamin Lee, M. D.;" "Remarks on a Modification of Taylor's Splint for Posterior Curvature of the Spine, by Benjamin Lee, M. D.," and "A New Prostatic Catheter."

